# Amalgam

**Published:** 1894-02

**Authors:** E. G. Clark

**Affiliations:** Portland, Oregon.


ARTICLE III.

### AMALGAM.

BY E. G. CLARK, D. D. S., PORTLAND, OREGON.

**Read before the Oregon State Dental Association, Oct. '93.**

The subject of this paper, viz: Amalgam, is one that might be called old and worn threadbare, by the discussion and the innumerable papers and dissertations that have been written for and against it.

However, considering the good that has been accomplished by its use in saving teeth, and the wanton destruction that has followed a bungling and defective manipulation and its improper use, it still is a subject of interest to the thinking, skillful dentist, and will be just so long as it holds the place it now occupies among the materials used by the practitioner in the filling of teeth. Knowing as we do that more teeth are filled with it to-day than any other material used, we should be skilled in the manipulation of it if we would accomplish beneficial and lasting results. It will not be necessary for me at this time to enter into a detailed description and discussion of amalgam, its history, the making of alloys or the merits of one alloy over another, for you all no doubt are familiar with the text books regarding the matter, and have read Flagg, Essig and others on the subject who have written concerning the manufacture of alloy. It will be my purpose to-night to direct your attention to another head.

Fillings of all descriptions fail because of certain different conditions, and the most prominent of these have been ably classed under four headings, viz: First, defective tissue; second, defective structure; third, abnormal surroundings, and fourth, defective manipulation. It is to the latter head that I will direct your attention in this paper, and shall try for a few moments to give to you my experience, limited as it may be, for what it is worth. Some one has aptly said that poets are born poets, and dentists must be born dentists. Some are so constituted that they can, seemingly without any material effort, work with a precision and nicety that many others can only attain by constant concentration, application and training, and there are others, I am pained to say, who are not born dentists, neither do they acquire even moderate manipulative ability, but go on without even trying to become more proficient, and use a slovenly, unscientific way of manipulation.

It has been said that the advent of vulcanite has brought about a degraded state of mechanical dentistry; on the other hand, why not say, and safely, too, that the unskillful use of amalgam has degraded operative dentistry? My reasons for this assertion are these: The dentist (so called) who prostitutes the profession by an ignorant and clumsy manipulation of vulcanite for dentures is also the one that brings abuse upon amalgam—by the defective way he fills teeth with it. He thinks he knows how to pack a cheap grade of rubber around sixty-five-cent or one-dollar teeth, and vulvanize them and turn them out for five, seven or ten dollars per set. He knows, or thinks he knows, how to excavate the caries out of a tooth and then "chuck in" (excuse the expression) a mass of cheap amalgam, without any preparation for dryness except the wiping of the cavity with a piece of cotton, and a piece of bibulous paper or a napkin held along the gum while he inserts his silver, as he terms it. Is it any wonder that these fillings fail, and patients come to us with a mouth full of black, disgusting monuments (no, not monuments, but ruins,) showing the ignorance of the man that placed them there, the wrong he has done and the havoc he has wrought? Those who do this kind of work should be eliminated from the profession and relegated to the rank in which he belongs, viz: with pick and axe or hoe and shovel, or something where they could be harmless and despoil no one's pocket-book. One great help to getting such emprics in the profession is caused by the pernicious habit of some persons styling themselves preceptors and turning students loose on an unsuspecting public after a few months as an apprentice in an office; but soon this will be stopped in this State, as one of the requirements of the board of dental examiners will be three years instead of two as a student in a reputable dental office before coming up for examination, or to hold a diploma from some recognized dental college.

Amalgam used properly and in indicated places is one of the great tooth conservers of the present day, and without it many a tooth that is now saved would be given over to the forceps. I am a friend to amalgam but a foe to a faulty manipulation of it. We find some of our numbers crying down amalgam and upholding gold for all places; but the number upholding this idea is not nearly so numerous as it was a few years ago. With these we must differ, for truly the conscientious dentist will be an eclectic and will use what is best indicated under the circumstances. In my own practice I have seen the folly of using gold for all places. Teeth that had been elaborately filled with it and a thing of beauty when finished, in a short time falling down and crumbling away, when if a good amalgam filling had been inserted with as much careful manipulation as had been expended on the elaborate gold filling the tooth would have been saved, standing in good order, giving comfort and satisfaction to the patient. Again, the more difficult the cavity to prepare, and the more inaccessible, in the same ratio does amalgam become the more valuable filling material and its use indicated because of its plasticity and easy introduction, and its general tooth preserving qualities that it has when handled and manipulated properly; and I assert without fear of contradiction that a good amalgam filling inserted under the above named conditions will be found faithful and doing good service when a gold filling under similar circumstances would have failed in a few years at farthest. Expertness in operating is just as necessary in the manipulation of amalgam as of gold or any other of our filling materials. It has been my experience to see some very faulty fillings with amalgam come from the hands of men whom I know to be competent gold manipulators. As to why this should occur I find but one reason, viz: they are prejudiced against amalgam and have a poor opinion of it in general, and reason in this manner (which by the way is not to their credit): "Oh, well, it is nothing but amalgam filling—won't last long—and I will get only two or three dollars for it anyway," so it is done hastily, the cavity illy prepared, moisture not fully removed, and, as a matter of course, it won't last long and a great wrong has been committed in taking even the two or three dollars. As to the fee, who says it should be two or three dollars? If you can successfully save a tooth with amalgam, or anything else, the pay should be commensurate to the service rendered, be it two, three, five or ten dollars. No wonder we hear the cry "Down with amalgam," especially when we find it manipulated by the hand of carelessness or ignorance—it is in poor company and utterly helpless.

The "sheet anchor" of modern operative dentistry as we all know is the rubber dam, and its use is just as imperative in the employment of amalgam as in the use of gold except in a few cases; but I wish to emphasize the fact, that in all cases of medium size, or extensive caries where considerable time has to be expended in the preparation of the cavity, our inflexible rule should be *apply the rubber dam*, and it is my rule of procedure to use the dam wherever and whenever I can. There may be some among you who will take issue with me as to the general use of the rubber dam and say that as good work can be done in excavating without its use as with. For the benefit of such I will say that the next time you have a medium-sized or large cavity to excavate and fill with amalgam, do your excavating primarily without the dam and then apply the rubber, and see what you will find, and I think that you will be convinced that the uses of the dam in all cases is good. It certainly will do no harm, but will be the means of causing a proper excavation of the cavity when if it had not been used you would have covered up some disorganized tissue, and you know the result.

Another great mistake that is often made is in not opening up the cavity and securing room to work. Even with this condition alone a cavity cannot be properly prepared, and one will be embarassed from the beginning to the end of the operation. *Always secure room to work as nearly direct as is possible*, also open so you can see into the cavity, remove all disorganized tissue, especially at the cervical border, and drill out stained or darkened fissures. Too often this is not done properly, and these are excellent places for caries to commence. We often see frail walls and ridges left. These should be cut away because of their easy fracture, and the general form of the cavity should be concave. The description of a properly prepared cavity for amalgam is given better by Dr. J. Foster Flagg and I will quote it verbatim: "In preparing cavities for amalgam the governing principles result in the making of cavities without angles, no flush walls, few if any pits, with cavity shape decidedly larger inside than out, with concave undercuts and largely overhanging edges; in short, he aims to make his cavity a concavity," all on account of the tendency of the amalgam to assume a sphereoidal shape, causing it to draw from angles and straight walls. He wants to shape the cavity so that he can place the amalgam just as he wants it to stay, so that bulging and crevicing are reduced to a minimum. The cavity being prepared, a coat of copal ether is given to the bottom and sides in teeth that are extremely sensitive. It is an excellent thing to use, and, in a great measure, prevents the shock from thermal changes.

In mixing the alloy one common mistake is the employment of too much mercury, and inserting the material in too plastic a state, which will have a tendency to cause the finished filling in the course of time to assume the spheriodal form and draw away from the edges much more so than if no excess had been present, thereby impairing the filling. The very best method of inserting amalgam filling as I find, is according to the method of Flagg termed wafering, with which no doubt you are all familiar. The final act in amalgam filling, and as thoroughly important as any step, is one that, alas ! we see too sadly neglected—the finishing of the filling, My experience is that fillings of amalgam that have been finished up, polished and burnished with as much care as those of gold, the results are far more complete and lasting. To sum it all up, we must remember to execute faithfully these six points : First, use the rubber dam; second, secure good access to the cavity; third, remove all defective tissue; fourth, form your cavity as nearly a concavity as possible; fifth, insert your filling by the wafering system; sixth, finish and burnish your filling at a second sitting. Then, I say, you will have amalgam work that you will be proud of and that your patients will appreciate.—*Pacific Dental Journal.*

